# LPS-induced inflammatory response is suppressed by Wnt inhibitors, Dickkopf-1 and LGK974

**DOI:** 10.1038/srep41612

**Published:** 2017-01-27

**Authors:** Jaewoong Jang, Yoonju Jung, Youngeun Kim, Eek-hoon Jho, Yoosik Yoon

**Affiliations:** 1 Department of Microbiology, Chung-Ang University College of Medicine, Seoul 156-756, Republic of Korea; 2 Department of Life Science, University of Seoul, Seoul, 130-743, Republic of Korea

## Abstract

In this study, LPS-induced inflammatory responses in BEAS-2B human bronchial epithelial cells and human umbilical vein endothelial cell (HUVEC)s were found to be prevented by Dickkopf-1 (DKK-1), a secreted Wnt antagonist, and LGK974, a small molecular inhibitor of the Wnt secretion. LPS-induced IκB degradation and NF-κB nuclear translocation as well as the expressions of pro-inflammatory genes including IL-6, IL-8, TNF- α, IL-1β, MCP-1, MMP-9, COX-2 and iNOS, were all suppressed by DKK-1 and LGK974 in a dose-dependent manner. The suppressive effects of LGK974 on NF-κB, IκB, and pro-inflammatory gene expression were rescued by ectopic expression of β-catenin, suggesting that the anti-inflammatory activity of LGK974 is mediated by modulation of the Wnt/β-catenin pathway and not by unrelated side effects. When Wnt recombinant proteins were treated to cells, Wnt3a and Wnt5a significantly induced pro-inflammatory gene expressions, while Wnt7a and Wnt10b showed little effects. It was also found that Wnt3a and Wnt5a expressions were significantly induced by LPS treatment. Consistently, knockdown of Wnt3a and Wnt5a blocked LPS-induced inflammatory responses, while treatment of recombinant Wnt3a and Wnt5a proteins rescued the inhibition of inflammatory responses by LGK974. Findings of this study showed that DKK-1 and LGK974 suppress LPS-induced inflammatory response by modulating Wnt/β-catenin pathway.

The Wnt/β-catenin pathway is known to regulate diverse biological processes including proliferation, differentiation, and development^1^. In the absence of Wnt, β-catenin is proteolyzed by the destruction complex containing glycogen synthase kinase 3β (GSK-3β), adenomatous polyposis coli (APC), and Axin. The binding of Wnt to frizzled (Fzd) and lipoprotein receptor-related protein 6 (LRP6) leads to phosphorylation of LRP6, which enhances the interaction between LRP6 and Axin. Consequently, the β-catenin destruction complex, which is composed of many proteins including APC, GSK3β and Axin, is disrupted, resulting in the stabilization and nuclear translocation of β-catenin^2^,^3^. Wnt antagonists include Frizzled-related protein, Cerberus, Wnt inhibitory factor, and Dickkopf-1 (DKK-1), among which DKK-1 prevents Wnt signaling by binding and inducing the internalization of LRP6 while others act by binding and sequestering Wnt^4^. During biosynthesis of Wnts, Wnts undergo posttranslational palmitoylation by porcupine (PORCN), a Wnt-specific acyltransferase that is required for Wnt secretion^5^.

Deregulation of the Wnt/β-catenin pathway has been described as a key player in the initiation, maintenance, progression, relapse and drug-resistance of many cancers^6^, and elevated levels of β-catenin, a hallmark of the activated Wnt/β-catenin pathway, have been observed in the most common human tumors^7^. Diverse chemical inhibitors of the Wnt pathway, including a Wnt secretion inhibitor, Fzd antagonist, Axin stabilizer, Dvl inhibitor and inhibitor of β-catenin/Tcf interaction, are being developed as anti-cancer drug candidates^6^. Among them, LGK974, a small-molecule inhibitor of PORCN which was developed as an anti-tumor drug candidate, has been shown to be effective in tumor models of murine breast cancer, human head and neck squamous cell carcinoma^8^ and glioblastoma^9^.

In our previous study, we found that β-catenin is involved in the inflammatory responses of LPS-stimulated BEAS-2B human bronchial epithelial cells^10^, and recently, it was reported that levels of Wnt5a and β-catenin were increased in LPS-stimulated BEAS-2B cells^11^. The detailed mechanism of LPS-induced activation of the Wnt/β-catenin pathway and the effects of Wnt pathway modulators, however, remain to be elucidated. In this study, LPS-induced inflammatory response was found to be prevented by DKK-1, a secreted Wnt antagonist. These results suggest that secreted Wnt may act via autocrine or paracrine fashion in LPS-induced inflammatory response, so we examined the effects of LGK974, a small molecular inhibitor of Wnt secretion, on LPS-induced inflammatory response.

## Results

### LPS-induced Wnt/β-catenin signaling was suppressed by a secreted Wnt antagonist, DKK-1

**Figure 1:**
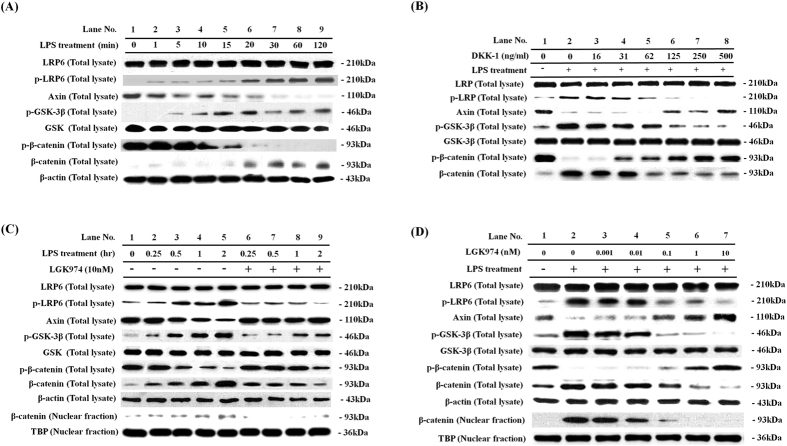
(**A**) Time course of LPS-induced activation of the WNT/β-catenin pathway. BEAS-2B human bronchial epithelial cells were stimulated with 0.1 μg/ml LPS for various time periods of 1–120 minutes. Western blotting was used to assess protein levels and phosphorylation of the members of the WNT/β-catenin pathway in total cell lysates. Beta-actin was used as endogenous control. **(B)** Suppressive effects of DKK-1 on LPS-induced activation of the WNT/β-catenin pathway. Cells were pretreated with 16–500 ng/ml of recombinant DKK-1 for 24 hours, followed by 0.1 μg/ml of LPS stimulation for 2 hours. **(C**,**D)** Suppressive effects of LGK974 on LPS-induced activation of the WNT/β-catenin pathway. Cells were pretreated with 10 nM LGK974 for 2 hours, followed by 0.1 μg/ml LPS stimulation for various time periods of 0.25–2 hours (**C**). Cells were pretreated with 0.001–10 nM of LGK974 for 2 hours, followed by 0.1 μg/ml of LPS stimulation for 2 hours (**D**).

Previously, we reported that β-catenin is involved in the inflammatory responses of LPS-stimulated BEAS-2B human bronchial epithelial cells^10^. In this study, we examined the mechanism for the regulation of β-catenin with an aim to determine targets for therapeutics. When BEAS-2B human bronchial epithelial cells were stimulated with 0.1 μg/ml of LPS for time periods ranging from 1 to 120 min, the phosphorylation of LRP6, a hallmark of initial Wnt pathway activation^12^, was elevated, while the protein levels of LRP6 did not change. The level of Axin, a negative regulator of the Wnt/β-catenin pathway, was found to be decreased. Phosphorylation of GSK-3β increased, while the protein levels of GSK-3β did not change. Phosphorylation of β-catenin decreased, while the levels of total β-catenin and nuclear β-catenin increased. These data clearly showed that the LPS treatment activates Wnt signaling at the level of LRP6 (Fig. 1). Since the noticeable increase of LRP6 phosphorylation occurred within 1–20 minutes of LPS treatment, we assumed that activation of Wnt signaling by LPS might not be controlled by transcriptional activation (Fig. 1A, lanes 2–6). The rapid responses suggested that LPS might control a posttranslational modification of protein(s) that can initiate Wnt signaling. We hypothesized that LPS might enhance Wnt ligand secretion or maturation. To test this hypothesis we tested whether the treatment of cells with DKK-1, a secreted Wnt antagonist that is well-known to induce the internalization of LRP6 on cell surface to prevent their association with Wnt ligands^13^, blocks LPS-induced Wnt signaling. When cells were pre-treated with 0 to 500 ng/ml of DKK-1 for 24 h and stimulated with 0.1 μg/ml of LPS for 2 h, LPS-induced phosphorylation of LRP6 was dose-dependently prevented by DKK-1 (Fig. 1B). LPS-induced reduction of Axin was also dose-dependently prevented by DKK-1. Phosphorylation of GSK-3β was decreased, and phosphorylation of β-catenin was increased, while the level of β-catenin was decreased by DKK-1 (Fig. 1B). These data clearly show that DKK-1 suppressed LPS-induced activation of the Wnt/β-catenin signaling in BEAS-2B human bronchial epithelial cells, and also suggest that the treatment of LPS induces secretion of Wnts, which may act via autocrine or paracrine fashion to induce Wnt/β-catenin signaling.

### LPS-induced Wnt/β-catenin signaling was suppressed by a porcupine inhibitor, LGK974

Since we showed that LPS-induced Wnt/β-catenin signaling was suppressed by a Wnt antagonist DKK-1, our data suggest a possibility that small molecules, which control the secretion of Wnts, may also suppress LPS-induced Wnt/β-catenin signaling. We therefore tested whether the treatment of LGK974, a porcupine (PORCN) inhibitor that blocks secretion of Wnt, suppressed LPS-mediated activation of Wnt signaling. Interestingly, pretreatment with 10 nM LGK974 before 2 hours of LPS stimulation suppressed the LPS-induced changes in phosphorylation of LRP6, GSK-3β, and β-catenin, as well as the changes in protein levels of Axin and β-catenin (Fig. 1C, lanes 6–9). Inhibition of the LPS-induced Wnt pathway by LGK974 was dose-dependent (Fig. 1D). Phosphorylation of LRP6, which was enhanced by treatment with LPS, was dose-dependently decreased by LGK974. The level of Axin, a negative regulator of the Wnt/β-catenin pathway, was dose-dependently rescued by LGK974. Increased GSK-3β phosphorylation by LPS treatment was dose-dependently blocked, while reduced phosphorylation of β-catenin was rescued. The level of β-catenin was dose-dependently decreased in total cell lysate, as well as in the nuclear fraction. These data clearly show that LGK974 suppressed LPS-induced Wnt/β-catenin signaling.

### DKK-1 and LGK974 suppressed LPS-induced inflammatory responses

**Figure 2:**
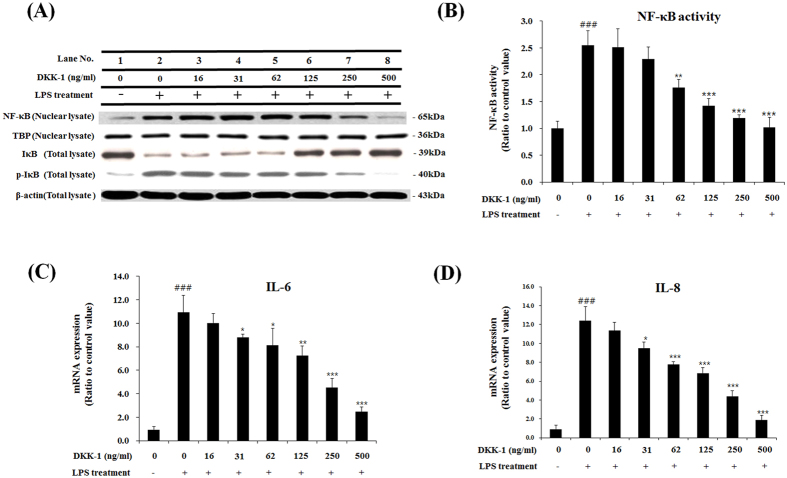
Suppressive effects of DKK-1 on LPS-induced inflammatory response in bronchial epithelial cells. BEAS-2B human bronchial epithelial cells were pretreated with 16–500 ng/ml of recombinant DKK-1 for 24 hours, followed by 0.1 μg/ml of LPS stimulation for 2 hours. Western blotting was used to assess protein levels and phosphorylation of NF-κB and IκB. Beta-actin and TBP were used as endogenous controls for total cell lysate and nuclear fraction, respectively (**A**). The binding of nuclear NF-κB for its target DNA sequence, 5′-GGGACTTTCC-3′, was measured by ELISA (**B**). The expressions of pro-inflammatory cytokine, IL-6 and IL-8, were measured by real time-qPCR (**C, D**). *p < 0.05, **p < 0.01, ***p < 0.001 compared with LPS-stimulated cells without DKK-1 pretreatment. ^###^p < 0.001 compared with cells without LPS stimulation.

Since our previous study showed that β-catenin is involved in LPS-induced inflammatory responses^10^, and DKK-1 and LGK974 inhibited LPS-induced Wnt signaling as shown in Fig. 1, we then tested whether DKK-1 or LGK974 blocks LPS-mediated inflammatory responses. It is well known that, during the LPS-induced inflammatory response, IκB is degraded upon phosphorylation which results in the consequent release and nuclear translocation of NF-κB^14^. When cells were stimulated with LPS, the level of NF-κB in the nuclear fraction increased, and the level of IκB decreased while the phosphorylation of IκB increased (Fig. 2A, lane 2). We found that DKK-1 dose-dependently decreases the nuclear NF-κB level, and increased the IκB level while decreasing phosphorylation of IκB (Fig. 2A, lanes 3–8). DKK-1 or LGK974 treatment alone had no effects on the levels of phospho-LRP6, Axin, phospho-β-catenin, total β-catenin, phospho-IκB and IκB as well as pro-inflammatory cytokine expression (Suppl Fig. 1). In order to quantify the effects of LGK974 on NF-κB activity, the binding of NF-κB to its target DNA sequence, 5′-GGGACTTTCC-3′, was measured. It was found that NF-κB activity was up-regulated by LPS stimulation and was suppressed by DKK-1 in a dose-dependent manner (Fig. 2B). As a result, LPS-induced expression of pro-inflammatory cytokines, IL-6 and IL-8, were inhibited by DKK-1 (Fig. 2C,D). Other pro-inflammatory genes including TNF-α, IL-1β, MMP-9, COX-2 and iNOS were also decreased by DKK-1 (Suppl Fig. 2) which showed that LPS-induced inflammatory response was suppressed by DKK-1.

**Figure 3:**
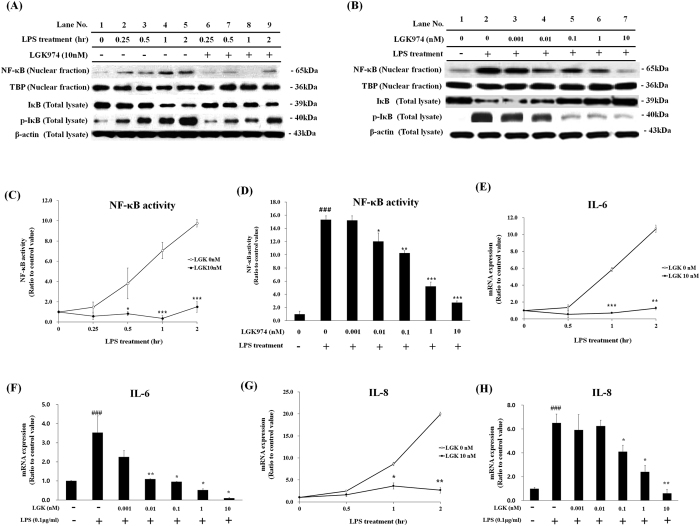
Suppressive effects of LGK974 on LPS-induced inflammatory response in bronchial epithelial cells. (**A**,**C**,**E**,**G**) BEAS-2B human bronchial epithelial cells were pretreated with 10 nM LGK974 for 2 hours, followed by 0.1 μg/ml LPS stimulation for various time periods of 0.25–2 hours. Western blotting was used to assess protein levels and phosphorylation of NF-κB and IκB. Beta-actin and TBP were used as endogenous controls for total cell lysate and nuclear fraction, respectively (**A**). The binding of nuclear NF-κB for its target DNA sequence, 5′-GGGACTTTCC-3′, was measured by ELISA (**C**). The expression of pro-inflammatory cytokines, IL-6 and IL-8, was measured by real time-qPCR (**E**,**G**). *p < 0.05, **p < 0.01, ***p < 0.001 compared with LPS-stimulated cells without LGK974 pretreatment at the same treatment hour. (**B**,**D**,**F**,**H**) Cells were pretreated with 0.001–10 nM of LGK974 for 2 hours, followed by 0.1 μg/ml of LPS stimulation for 2 hours. Western blotting was used to assess protein levels and phosphorylation of NF-κB and IκB (**B**). The binding of nuclear NF-κB for its target DNA sequence was measured by ELISA (**D**). The expression of pro-inflammatory cytokines was measured by real time-qPCR (F, H). *p < 0.05, **p < 0.01, ***p < 0.001 compared with LPS-stimulated cells without LGK974 pretreatment. ^###^p < 0.001 compared with cells without LPS stimulation pretreatment.

We then tested whether LGK974 can also suppress the LPS-induced inflammatory response. Pretreatment with 10 nM LGK974 suppressed all LPS-induced changes in NF-κB and IκB (Fig. 3A). These suppressive effects of LGK974 were dose-dependent. NF-κB level in nuclear fraction was dose-dependently decreased by LGK974. IκB level was dose-dependently increased when phosphorylation of IκB decreased (Fig. 3B). Target DNA binding activity of NF-κB was also significantly and dose-dependently decreased by LGK974 (Fig. 3C,D). When cells were stimulated with LPS for various time periods, the expression of pro-inflammatory cytokines, IL-6 and IL-8, was up-regulated; however, this increased expression was significantly inhibited by 10 nM LGK974. The inhibition of IL-6 and IL-8 expression by LGK974 was dose-dependent (Fig. 3E–H). The expressions of other pro-inflammatory genes including TNF-α, IL-1β, MMP-9, COX-2 and iNOS were similarly suppressed by LGK974 (Suppl Fig. 3). Overall, these data suggest that LGK974 suppresses LPS-induced inflammatory response by inhibiting NF-κB signaling.

**Figure 4:**
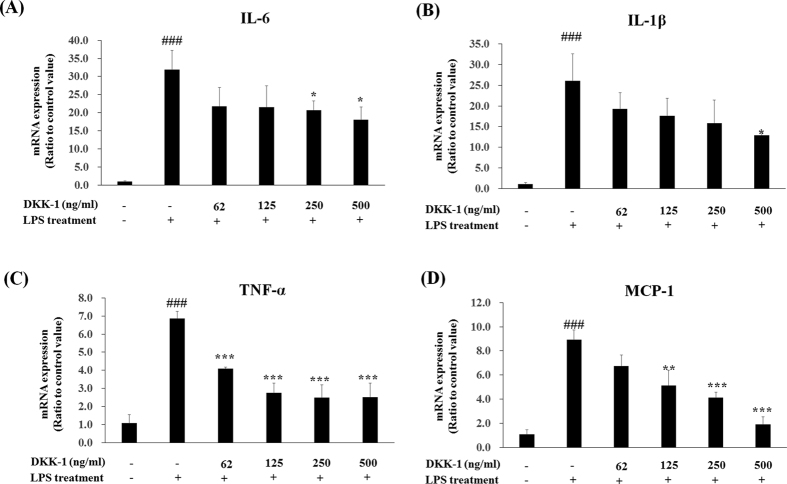
Suppressive effects of DKK-1 on LPS-induced inflammatory responses in HUVECs. HUVECs were pretreated with 62–500 ng/ml of recombinant DKK-1 for 24 hours followed by 0.1 μg/ml of LPS stimulation for 4 hours. The expressions of pro-inflammatory cytokines were measured by real time-qPCR. *p < 0.05, **p < 0.01, ***p < 0.001 compared with LPS-stimulated cells without DKK-1 pretreatment. ^###^p < 0.001 compared with cells without LPS stimulation.

**Figure 5:**
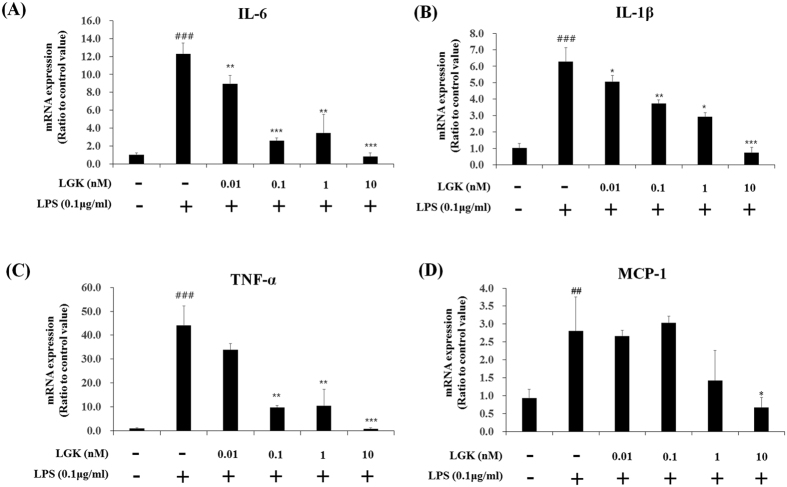
Suppressive effects of LGK974 on LPS-induced inflammatory responses in HUVECs. HUVECs were pretreated with 0.01–10 nM of LGK974 for 2 hours, followed by 0.1 μg/ml of LPS stimulation for 4 hours. The expressions of pro-inflammatory cytokines were measured by real time-qPCR. *p < 0.05, **p < 0.01, ***p < 0.001 compared with LPS-stimulated cells without DKK-1 or LGK974 pretreatment. ^###^p < 0.001 compared with cells without LPS stimulation.

We tested whether DKK-1 and LGK974 also suppressed inflammatory responses in other cell types, especially primary cells, and human umbilical vein endothelial cell (HUVEC)s obtained from pooled donners were tested. Anti-inflammatory effects of DKK-1 or LGK974 in LPS-stimulated HUVECs were similar as shown in BEAS-2B human bronchial epithelial cells (Figs 4 and 5). LPS-induced pro-inflammatory cytokine expressions were suppressed by DKK-1 and LGK974 significantly and dose-dependently. These data showed that DKK-1 and LGK974 exert anti-inflammatory effects in endothelial cells as well as epithelial cells.

### The suppressive effects of LGK974 on the LPS-induced inflammatory response were mediated by the regulation of Wnt/β-catenin signaling

**Figure 6:**
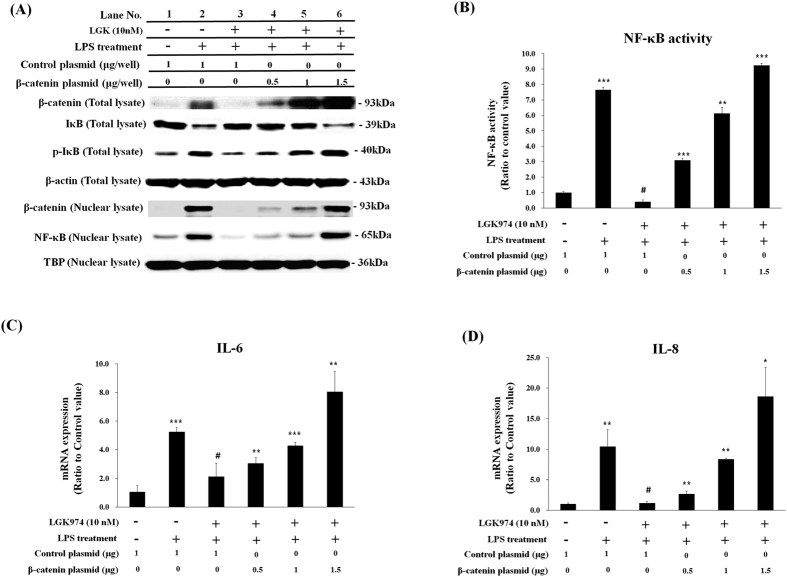
Rescuing effects of β-catenin expression on anti-inflammatory activity LGK974. BEAS-2B human bronchial epithelial cells were transfected with 0.5 to 1.5 μg/well of β-catenin expression plasmid or 1.0 μg/well of control plasmid one day before pretreatment with 10 nM LGK974 for 2 hours, followed by 0.1 μg/ml LPS stimulation for 2 hours. Total cell lysates and nuclear fractions were analyzed by Western blotting for protein levels and phosphorylation. Beta-actin and TBP were used as endogenous controls for total cell lysates and nuclear fractions, respectively (**A**). The binding of nuclear NF-κB for its target DNA sequence, 5′-GGGACTTTCC-3′, was measured by ELISA (**B**). The expression of pro-inflammatory cytokine, IL-6 and IL-8, were measured by real time-qPCR (**C**,**D**). Data were compared to those of cells transfected with control plasmid and treated with LGK974 and LPS treatment (#-marked column). *p < 0.05, **p < 0.01, ***p < 0.001

We then tested whether the suppression of LPS-induced inflammatory responses by LGK974 was due to the regulation of Wnt signaling. When cells were transfected with β-catenin expressing plasmid one day before LGK974 treatment and LPS stimulation, LPS-induced increases of nuclear NF-κB and phospho-IκB levels (Fig. 6A, lane 2) were suppressed by LGK974 (Fig. 6A, lane 3) and were dose-dependently rescued by β-catenin overexpression (Fig. 6A, lanes 4–6). The LPS-induced decrease in IκB (Fig. 6A, lane 2) was suppressed by LGK974 (Fig. 6A, lane 3), and this was dose-dependently rescued by β-catenin expression (Fig. 6A, lanes 4–6). Target DNA binding activity of NF-κB showed an identical pattern with nuclear NF-κB level (Fig. 6B). The LPS-induced up-regulation of IL-6 and IL-8 was suppressed by LGK974 and was dose-dependently rescued by β-catenin expression similar to nuclear NF-κB level and its DNA binding activity (Fig. 6C,D). Expressions of other pro-inflammatory genes including TNF-α, IL-1β, MMP-9, COX-2 and iNOS were similarly rescued by β-catenin (Suppl Fig. 4). Overall, these data suggest that anti-inflammatory effects of LGK974 are due to the reduced level of β-catenin.

**Figure 7:**
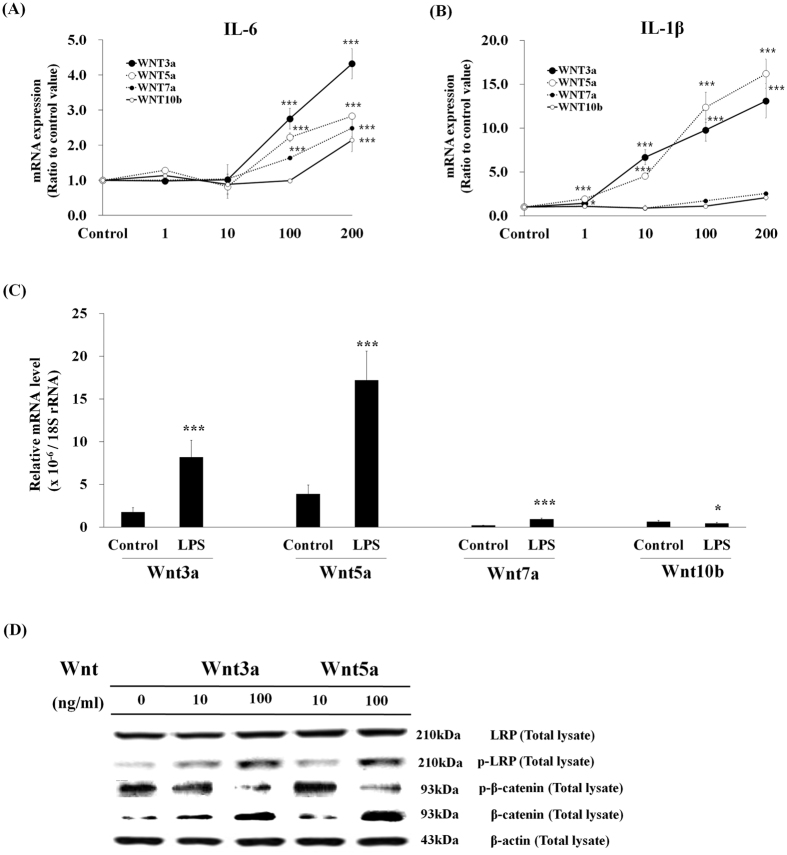
Effects of recombinant Wnt protein on pro-inflammatory cytokine expression. **(A**,**B)** BEAS-2B human bronchial epithelial cells were treated with 1–200 ng/ml of recombinant Wnt3a, Wnt5a, Wnt7a or Wnt10b protein for 2 h. The expressions of pro-inflammatory genes were measured by real time-qPCR. Comparison of the expression levels of Wnts in control cells and LPS-stimulated cells **(C)**. BEAS-2B human bronchial epithelial cells were stimulated with none or 0.1 μg/ml LPS for 2 hr. The relative mRNA expression levels of Wnt3a, Wnt5a, Wnt7a and Wnt10b in control cells and LPS-treated cells were measured by real time-qPCR. *p < 0.05, **p < 0.01, ***p < 0.001 compared with control cells. Activation of the WNT/β-catenin pathway by recombinant Wnt proteins **(D)**. BEAS-2B human bronchial epithelial cells were stimulated with 10 or 100 ng/ml of recombinant Wnt3a or Wnt5a proteins for 2 hr. Western blotting was used to assess protein levels and phosphorylation of the members of the WNT/β-catenin pathway in total cell lysates. Beta-actin was used as an even loading control. Each Blot was cropped at the position of the blotted protein and high-contrast was not used.

To find which Wnts are involved in the inflammatory responses, commercially available Wnt recombinant proteins were treated to BEAS-2B human bronchial epithelial cells, and it was found that Wnt3a and Wnt5a induced the expression of pro-inflammatory cytokines, while Wnt7a and Wnt10b showed little effects (Fig. 7A and B). Realtime-qPCR analysis showed that the basal levels of Wnt3a and Wnt5a were high, whereas those of Wnt7a and Wnt10b were low, and the expressions of Wnt3a and Wnt5a were increased by LPS treatment in BEAS-2B human bronchial epithelial cells (Fig. 7C), suggesting that Wnt3a and Wnt5a are involved in LPS-induced inflammatory responses. When cells were treated with recombinant Wnt3a and Wnt5a, phosopho-LRP6 was increased and phospho-β-catenin was decreased while total β-catenin was increased, showing that canonical Wnt/β-catenin pathway was activated by both Wnts (Fig. 7D).

**Figure 8:**
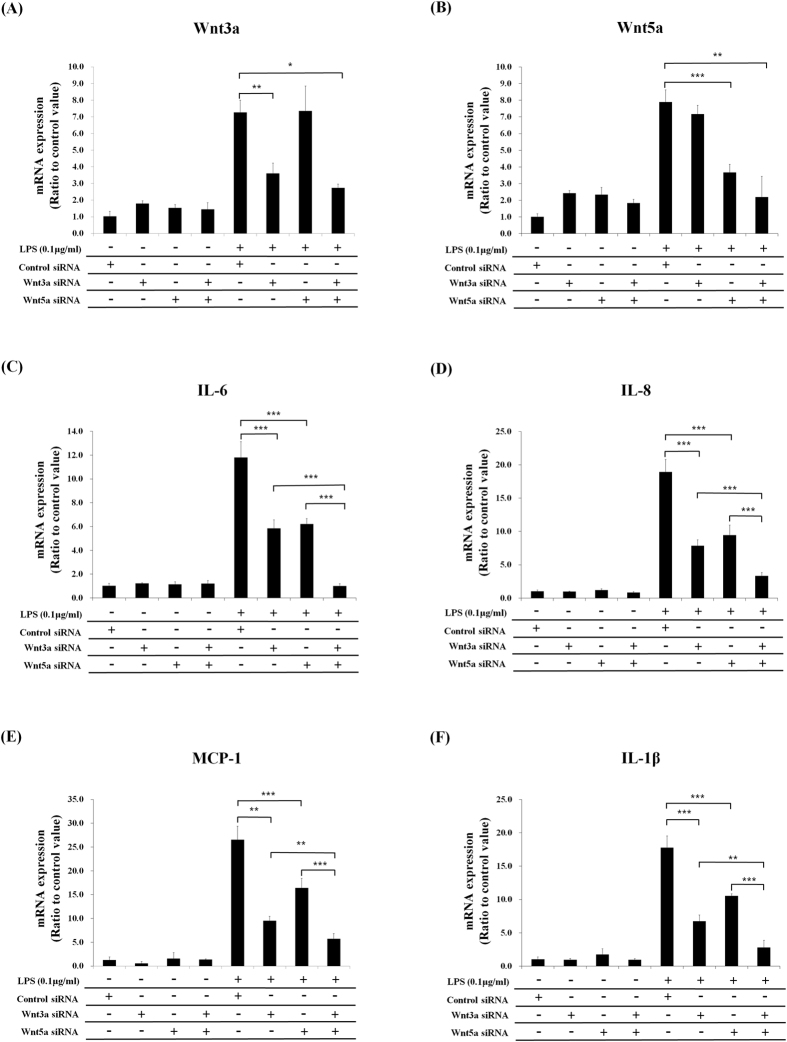
Suppressive effects of Wnt3a and Wnt5a knockdown on LPS-induced pro-inflammatory cytokine expressions. BEAS-2B cells were transfected with Wnt3a siRNA, Wnt5a siRNA or both followed by 0.1 μg/ml of LPS stimulation for 2 hours. The expressions of Wnts and pro-inflammatory cytokines were measured by real time-qPCR. *p < 0.05, **p < 0.01, ***p < 0.001.

**Figure 9:**
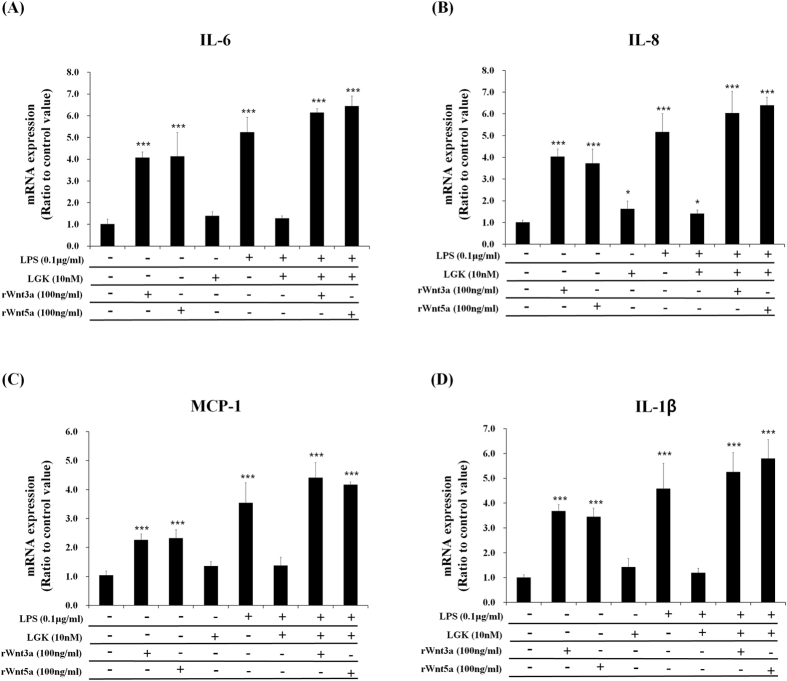
Rescuing effects of recombinant Wnt3a and Wnt5a on anti-inflammatory activity LGK974. BEAS-2B human bronchial epithelial cells were pretreated with 10 nM LGK974 for 2 hours, followed by 0.1 μg/ml LPS stimulation for 2 hours without or with 100 ng/ml of recombinant Wnt 3a or Wnt5a protein. The expressions of pro-inflammatory genes were measured by real time-qPCR. Data were compared to untreated cells. *p < 0.05, **p < 0.01, ***p < 0.001 compared with untreated cells.

To prove the involvement of Wnt3a and Wnt5a in LPS-induced inflammatory response, we performed knockdown experiments using their siRNAs, and found that LPS-induced pro-inflammatory cytokine expressions were significantly suppressed by the knockdown of Wnt3a or Wnt5a. The double knockdown has synergistic effects for the inhibition of inflammatory responses compared with single knockdown of Wnt3a or Wnt5a (Fig. 8). When recombinant Wnt3a or Wnt5a protein was added to LGK974-pretreated and LPS-stimulated BEAS-2B human bronchial epithelial cells, the suppressive effects of LGK974 on LPS-induced pro-inflammatory cytokine expressions were rescued (Fig. 9). Overall, these data strongly suggest that LGK974-mediated inhibition of inflammatory response by LPS is mediated by the blocking of secretion and expression of Wnts, especially Wnt3a and Wnt5a.

## Discussion

LGK974, an inhibitor of the Wnt secretion, has been developed as an anti-cancer drug candidate. In this study, however, we report the anti-inflammatory activity of LGK974, which has not been reported until now. Our interest in LGK974 originated from the finding that LPS-induced inflammatory response was suppressed by DKK-1, a secreted Wnt antagonist which prevents the binding of Wnt with LRP6 coreceptor on cell surface (Fig. 2). These results suggested that secreted Wnt may act via autocrine or paracrine fashion in LPS-induced inflammatory response and an inhibitor of Wnt secretion may have anti-inflammatory activity. LGK974 is a small molecular inhibitor of PORCN, which plays an essential role in posttranslational palmitoylation of Wnt ligands required for their secretion. LGK974 has been reported to inhibit the over-activated Wnt signaling in cancer cells^8^, but our data showed that LGK974 suppressed the Wnt signaling that is activated during LPS-induced inflammatory response (Figs 1C,D and 3). These data suggest that the secretion of Wnt and its binding to LRP6 coreceptor, in an autocrine or paracrine fashion, is required to induce LPS-induced pro-inflammatory response.

Compared with untreated cells, LPS-stimulated cells showed higher pro-inflammatory cytokine expressions mediated by an elevated nuclear NF-κB level, which was significantly suppressed by LGK974 in a dose-dependent manner (Fig. 3). Our data show that DKK-1 and LGK974 exert anti-inflammatory activities in LPS-stimulated endothelial cells as well as epithelial cells (Figs 4 and 5). However, when DKK-1 or LGK974 were co-treated with LPS at the same time, suppressive effects were minimal or not observed compared with pre-treatment (Suppl Fig. 1). DKK-1 induces the internalization of LRP6^15^ and LGK974 inhibits porcupine, a transmembrane protein located on the endoplasmic reticulum^16^, while LPS acts on cell surface Toll-like receptor 4^17^. The action of LPS may be much more rapid than that of DKK-1 or LGK974, and if they are treated at the same time, LPS may induce pro-inflammatory signaling before DKK-1 and LGK974 exerts their inhibitory effects. However, in a situation where LPS is generated from infected bacteria, DKK-1 or LGK974 may have some preventive effects on tissue damage mediated by over-expressed pro-inflammatory genes such as MMP, COX-2, iNOS as well as pro-inflammatory cytokines.

To show that the anti-inflammatory activity of LGK974 was mediated by its modulation of the Wnt/β-catenin pathway and not by an unrelated side effect, we analyzed the effects of β-catenin expression on the anti-inflammatory activity of LGK974. It was shown that the anti-inflammatory activity of LGK974 on NF-κB/IκB levels and pro-inflammatory cytokine expression was dose-dependently rescued by β-catenin expression (Fig. 6). Our data rule out the possibility that the anti-inflammatory activity of LGK974 is a side effect not related to the Wnt/β-catenin pathway.

In this study, both Wnt3a and Wnt5a were found to activate canonical Wnt/β-catenin pathway, which was shown by the up-regulations of phosopho-LRP6 and total β-catenin as well as the reduction of phospho-β-catenin (Fig. 7D). Wnt3a has been well known to induce canonical Wnt signaling^18^. Wnt5a has been classified as a non-canonical Wnt family member, however, it was found that Wnt5a can also activate canonical β-catenin signaling in the presence of the appropriate Fzd receptor^19^, and it was also reported that Wnt5a induced canonical signaling in LPS-treated BEAS-2B bronchial epithelial cells, the same experimental condition used in this study^11^.

It was also found that Wnt3a and Wnt5a mRNA expressions were significantly induced 2 hr after LPS treatment (Figs 7C and 8A,B). Wnt3a induction in LPS-induced inflammatory response is the new finding of this study, but Wnt5a induction has been previously reported. It was reported that LPS induced Wnt5a mRNA in THP-1 human monocytic cells at 4 hr after stimulation, which were inhibited by NF-κB inhibitors suggesting that NF-κB is involved in Wnt5a expression^20^. The expression of Wnt5a required Toll-like receptor signaling and NF-κB activation in human macrophages following stimulation with mycobacteria, demonstrating that Wnt5a was up-regulated through NF-κB activation^21^. It was also reported that Wnt5a activate NF-κB via Wnt5a-Fzd5-NFκB circuit, and Wnt5a-mediated NF-κB homeostasis sustains Wnt5a expression in a feed-forward mode to sustain immune response of macrophage^22^. In this study, LPS-induced inflammatory response did not take place when Wnt signaling is disrupted by LGK974 or Wnts knockdown. This means that LPS-induced inflammatory response requires active Wnt signaling. LPS also significantly induced Wnt3a and Wnt5a expressions which may result in the amplification of Wnt signaling, suggesting a feed-forward relation of Wnt signaling and inflammatory signaling for the maintenance and amplification of inflammatory response.

The bronchial epithelium induces inflammatory responses through the production of pro-inflammatory cytokines, the suppression of which can be of important therapeutic value for various respiratory diseases^23^. Sepsis, a systemic inflammation in response to circulating microbes or microbial toxin such as LPS, is the most common factor for acute respiratory distress syndrome with high morbidity and mortality^24^. The bronchial epithelium is usually exposed to environmental Gram-negative bacteria, which contain the major virulence factor LPS in their cell walls^25^. Up to 1 μg/m^3^ of airborne LPS has been found in various environments^26^. It is well-known that LPS induces expression pro-inflammatory cytokines, including IL-6 and IL-8, in bronchial epithelial cells^27^, and that this is mediated by the activation of NF-κB^28^. Many previous studies have suggested that the modulation of NF-κB activity is crucially important for effectively treating respiratory diseases involving pulmonary inflammation, because NF-κB plays a major role in the inflammatory responses of bronchial epithelial cells to various pathogens and toxic environmental factors such as bacteria, viruses, dust mite allergen, cigarette smoke, diesel particles, and dust^29^,^30^,^31^,^32^,^33^,^34^. The inflammatory response of endothelial cells is also of critical importance in various diseases such as septic shock, anaphylactic shock, thrombosis, atherosclerosis and chronic venous insufficiency, and controlling inflammation at the level of endothelial cells may efficiently retard or reverse these pathogenic processes^35^. HUVECs have been widely used as *in vitro* experimental models for vascular inflammation, and especially, LPS-stimulated HUVECs have been used as an *in vitro* model of sepsis^36^,^37^. In this study, LPS-induced pro-inflammatory cytokine expressions in HUVECs were significantly suppressed by DKK-1 or LGK974, suggesting that their anti-inflammatory effects may be useful for the treatment of vascular inflammation and sepsis.

In this study, we provide evidence that Wnt inhibitors, DKK-1 and LGK974, have anti-inflammatory effects in LPS-stimulated epithelial cells and endothelial cells, suggesting that they can be used to suppress diverse pro-inflammatory conditions.

## Methods

### Cell culture and Reagents

BEAS-2B human bronchial epithelial cells from American Type Culture Collection (Manassas, VA, USA) were cultured in Dulbecco’s modified Eagle’s medium (DMEM) supplemented with 10% fetal bovine serum, 100 μg/mL streptomycin, and 100 units/mL penicillin at 37 °C in a 5% CO_2_ atmosphere. Human umbilical vein endothelial cell (HUVEC)s, obtained from pooled donners and cryopreserved at passage 1, were purchased from Lonza (Walkersville, MD, USA) and cultured in EGM-2 endothelial basal medium with growth supplements (Lonza) and 2% fetal bovine serum.

Anti-IκB, anti-phospho IκB, anti-NF-κB p65, anti-GSK-3β, anti-phospho GSK-3β, anti-phospho β-catenin, and anti-phospho LRP6 (Ser 1490) primary and secondary antibodies were obtained from Cell Signaling Inc. (Beverly, MA, USA). Anti-LRP6 antibody was obtained from Abcam (Cambridge, MA, USA), and anti-β-catenin antibody was purchased from BD Transduction Laboratories Inc. (Lexington, KY, USA). Anti-Axin, anti-β-actin, and anti-TATA box binding protein (TBP) antibodies were purchased from Santa Cruz Biotechnology Inc. (Santa Cruz, CA, USA). Recombinant human Dickkopf-1 (DKK-1) and recombinant human Wnt3a, Wnt5a, Wnt7a and Wnt10b proteins were from R&D systems (Minneapolis, MN, USA). LPS from *Klebsiella pneumoniae* was obtained from Sigma-Aldrich (St. Louis, MI, USA).

### Preparation of total cell lysate and Western blotting

Cells were lysed using ice-cold RIPA buffer containing 25 mM Tris–HCl (pH 7.6), 150 mM NaCl, 1% Nonidet P-40, 1% sodium deoxycholate, 0.1% SDS, and protease inhibitor cocktail (Sigma-Aldrich). Total cell lysates were obtained after removing the insoluble materials by centrifugation at 20,000 × g for 20 minutes at 4 °C. The protein concentrations were determined using a BCA protein assay kit (Pierce, Rockford, IL, USA), and 50 μg of proteins were separated by 12% polyacrylamide gel electrophoresis and transferred to nitrocellulose membranes at 150 mA for 1.5 hours. The membranes were then blocked for two hours at room temperature with phosphate buffered saline containing 5% skim milk and 0.1% Tween 20. They were then incubated with 1:1000 dilutions of primary antibody overnight at 4 °C and subsequently with a 1:1000-dilution of horseradish peroxidase-conjugated secondary antibody for two hours at room temperature. Peroxidase activity was visualized using an ECL kit (Bio-Rad Laboratories Inc., USA). Beta-actin was used as a loading control.

### Analysis of nuclear β-catenin and NF-κB levels

Cells were harvested, and nuclear fractions were obtained using a nuclear extract kit (Active Motif, Carlsbad, CA, USA). Protein concentrations of the nuclear fractions were measured using a BCA protein assay kit (Pierce). Ten micrograms of nuclear protein were separated by 12% polyacrylamide gel electrophoresis to be analyzed by Western blotting using an anti-β-catenin antibody or anti-NF-κB p65 antibody. Anti-TATA box binding protein (TBP) antibody (Santa Cruz Biotechnology Inc.) was used as a loading control for the nuclear extract.

### Beta-catenin over expression and Wnt knock-down

Cells were seeded in a 12-well plate at a density of 5 × 10^5^ cells/well and, on the following day, were transfected with control pQNCXII plasmid or β-catenin expression plasmid^38^, using lipofectamine 2000 transfection reagent (Invitrogen, Carlsbad, CA, USA). After 24 hours, cells were pretreated with 10 nM LGK974 for two hours and subsequently stimulated with 0.1 μg/ml LPS for 2 hours. For knock-down experiments, cells were transfected with 50 nM of control siRNA and siRNA for Wnt3a or 5a (Santa Cruz Biotechnology, Inc.) using Lipofectamine RNAiMAX transfection reagent (Invitrogen, Carlsbad, CA, USA).

### Measurement of pro-inflammatory cytokine expression

Total RNA was extracted from cells grown in a 12-well plate using an RNeasy kit (Qiagen, Hilden, Germany). Total RNA was reverse-transcribed using a cDNA Reverse Transcription Kit (Applied Biosystems, Inc., Foster City, CA). Reverse transcription reaction was performed in a final volume of 20 μL, which included 100 mM of dNTP, random primers, MultiScribe™ Reverse Transcriptase, RNase inhibitor, and 1 μg of total RNA. The reaction mixtures were heated at 25 °C for 10 minutes, 37 °C for 120 minutes, and 85 °C for five seconds. Real-Time quantitative PCR (RT-qPCR) was performed using a Step One PCR System (Applied Biosystems) in a final volume of 20 μL, which included TaqMan gene expression master mix, an optimized concentration of each primer, 250 nM of a TaqMan probe, and 2 μL of cDNA reaction mix. The reaction mixtures were preheated at 95 °C for 10 minutes to activate the enzyme and then underwent 40 cycles of melting at 95 °C for 15 seconds and annealing/extension at 60 °C for one minute. The efficiencies of RT qPCRs were about 100%. The assay-on-demand gene expression products (Applied Biosystems, Inc.) were used to measure the mRNA expression levels of IL-6 (Hs00174131_m1), IL-8 (Hs99999034_m1), TNF-α (Hs01113624_g1), IL1-β (Hs01555410_m1), MCP-1 (Hs00234140/m1), MMP-9 (Hs00234579_m1), COX-2 (Hs01075529_m1), iNOS (Hs00153133_m1) and 18 S rRNA (Hs99999901_s1). 18 S rRNA was used as an internal control. The mRNA levels for each sample were normalized against the 18 S rRNA level. The ratios of normalized mRNA to control sample were determined using the comparative Ct method^39^.

### NF-κB activity assay for target DNA binding

Nuclear fractions were prepared from LPS-treated cells using a nuclear extract kit (Active Motif, Carlsbad, CA, USA), and protein concentrations were measured using a BCA protein assay kit (Pierce, Rockford, IL, USA). To assess the binding of NF-κB to its target DNA sequence (5′-GGGACTTTCC-3′), NF-κB activity was assayed using a TransAM NF-κB ELISA kit (Active Motif). Ten micrograms of protein from the nuclear fractions were added to a 96-well plate coated with oligonucleotide, 5′-GGGACTTTCC-3′. After incubation and washing, an anti-NF-κB primary antibody was added to the wells, followed by horseradish peroxidase-conjugated secondary antibody.

### Statistical analysis

All data were expressed as mean ± standard deviation of at least three replicate experiments. Statistically significant differences between treated and untreated samples were detected using unpaired t-tests. All analyses were performed using SPSS ver. 14 (SPSS, Chicago, IL, USA).

## Additional Information

**How to cite this article**: Jang, J. *et al*. LPS-induced inflammatory response is suppressed by Wnt inhibitors, Dickkopf-1 and LGK974. *Sci. Rep.* **7**, 41612; doi: 10.1038/srep41612 (2017).

**Publisher's note:** Springer Nature remains neutral with regard to jurisdictional claims in published maps and institutional affiliations.

## Supplementary Material

Supplementary Figures
