# Supplementary material for: LPS-induced inflammatory response is suppressed by Wnt inhibitors, Dickkopf-1 and LGK974
Source: Sci Rep. 2017 Jan 27;7:41612. doi: 10.1038/srep41612 (PMC5269682; doi:10.1038/srep41612)
Supplement: Supplementary Figures [file srep41612-s1.pdf]

Supplementary Information of :

LPS-induced inflammatory response is suppressed by Wnt inhibitors, Dickkopf-1 and LGK974

Jaewoong Jang[1], Yoonju Jung[1], Youngeun Kim[2], Eek-hoon Jho[2*], Yoosik Yoon[1*]

[1] Department of Microbiology, Chung-Ang University College of Medicine, Seoul 156-756, Republic of Korea

[2] Department of Life Science, University of Seoul, Seoul, 130-743, Republic of Korea

* ej70@uos.ac.kr (E-h. Jho) and thanks@cau.ac.kr (Y. Yoon)

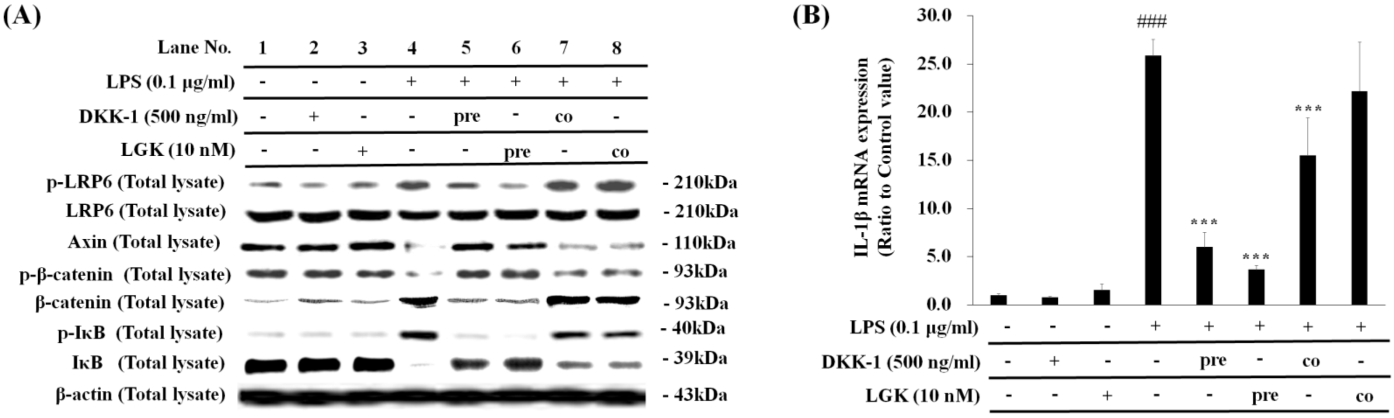

Supple Fig. 1. **Effects of DKK-1 and LGK974 alone and co-treatment with LPS on Wnt signaling and pro-inflammatory response.**

BEAS-2B human bronchial epithelial cells were treated with none (lane 1), DKK-1 alone (lane 2), LGK974 alone (lane 3), LPS alone (lane 4), LPS+DKK-1 pretreatment (lane 5), LPS+LGK974 pretreatment (lane 5), LPS+DKK-1 co-treatment (lane 7) and LPS+LGK974 co-treatment (lane 8). The protein levels of phospho-LRP6, total LRP6, Axin, phospho-β-catenin, total β-catenin, phospho-IκB and IκB were analyzed by Western blot (A). The mRNA expression of pro-inflammatory cytokine IL-1β were measured by realtime-qPCR (B). * $p<0.05$, ** $p<0.01$, *** $p<0.001$ compared with cells treated with LPS alone. ### $p<0.001$ compared with cells treated with none.

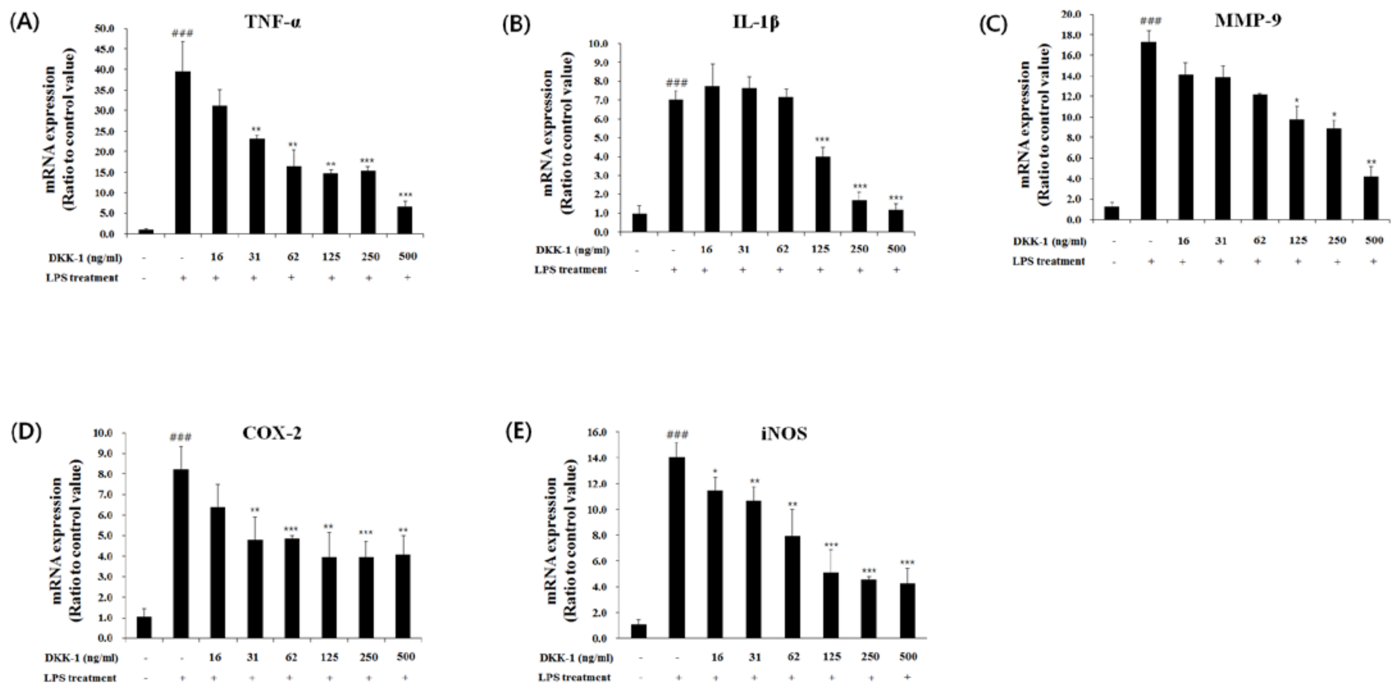

Suppl Fig. 2 Suppressive effects of DKK-1 on pro-inflammatory gene expression.

BEAS-2B human bronchial epithelial cells were pretreated with 16-500 ng/ml of recombinant DKK-1 for 24 hours, followed by 0.1 μg/ml of LPS stimulation for 2 hours. The expression of pro-inflammatory genes including TNF-α, IL-1β, MMP-9, COX-2 and iNONS was measured by real time-qPCR. * $p<0.05$, ** $p<0.01$, *** $p<0.001$ compared with LPS-stimulated cells without DKK-1 pretreatment. ### $p<0.001$ compared with cells without LPS stimulation.

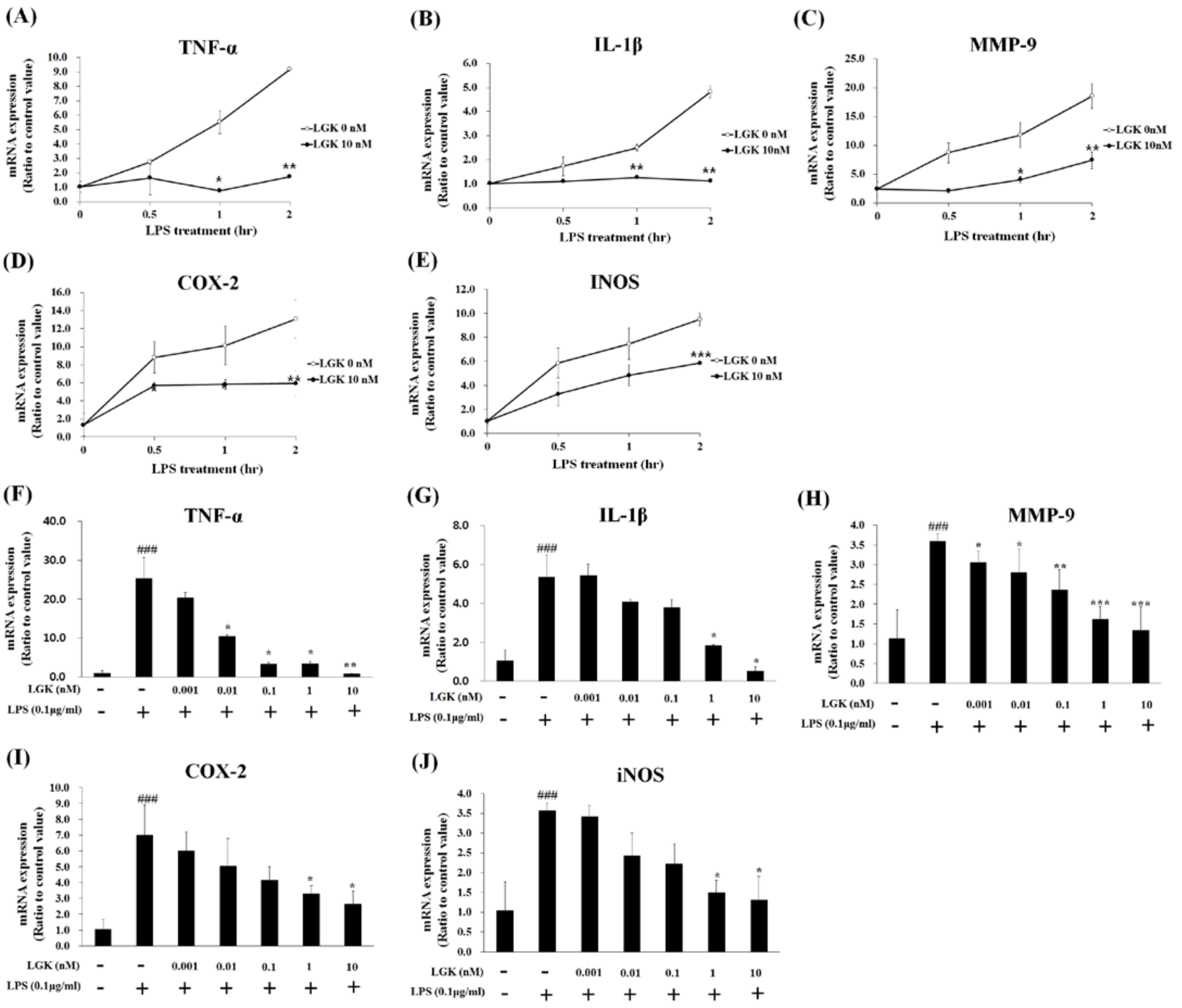

Suppl Fig. 3 **Suppressive effects of LGK-974 on pro-inflammatory gene expression.**

(A-E) BEAS-2B human bronchial epithelial cells were pretreated with 10 nM LGK974 for 2 hours, followed by 0.1 µg/ml LPS stimulation for various time periods of 0.25–2 hours. The expression of pro-inflammatory genes including TNF-α, IL-1β, MMP-9, COX-2 and iNONS was measured by real time-qPCR. * $p<0.05$, ** $p<0.01$, *** $p<0.001$ compared with LPS-stimulated cells without LGK974 pretreatment at the same treatment hour.

(F-J) Cells were pretreated with 0.001 - 10 nM of LGK974 for 2 hours, followed by 0.1 µg/ml of LPS stimulation for 2 hours. The expression of pro-inflammatory genes was measured by real time-qPCR. * $p<0.05$, ** $p<0.01$, *** $p<0.001$ compared with LPS-stimulated cells without LGK974 pretreatment. ### $p<0.001$ compared with cells without LPS stimulation.

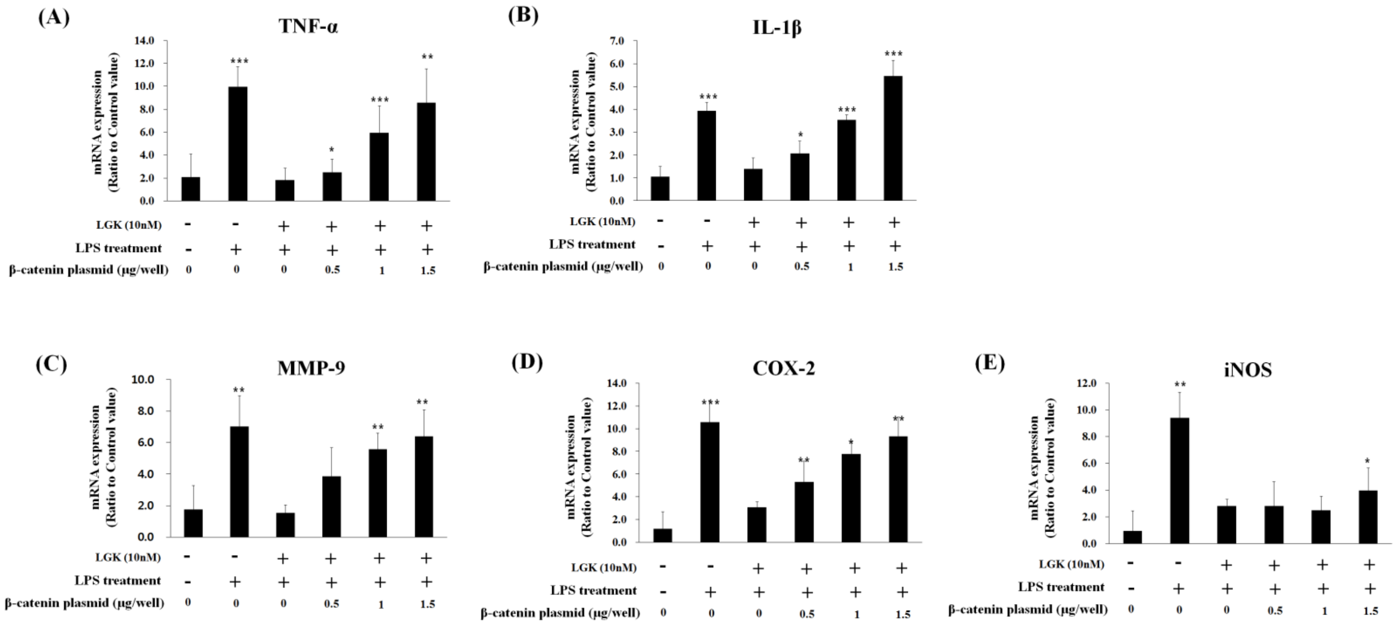

Suppl Fig. 4 Rescuing effects of β-catenin expression on pro-inflammatory gene expression suppressed by LGK974.

BEAS-2B human bronchial epithelial cells were transfected with 0.5 to 1.5 μg/well of β-catenin expression plasmid one day before pretreatment with 10 nM LGK974 for 2 hours, followed by 0.1 μg/ml LPS stimulation for 2 hours. The expression of pro-inflammatory genes including TNF-α, IL-1β, MMP-9, COX-2 and iNONS was measured by real time-qPCR. Data were compared to those of cells transfected with 0 μg of β-catenin plasmid with LGK974 and LPS treatment (#-marked column). * $p<0.05$, ** $p<0.01$, *** $p<0.001$
